# Comparative cost analysis of cervical cancer screening programme based on molecular detection of HPV in Spain

**DOI:** 10.1186/s12905-021-01310-8

**Published:** 2021-04-26

**Authors:** R. Ibáñez, M. Mareque, R. Granados, D. Andía, M. García-Rojo, J. C. Quílez, I. Oyagüez

**Affiliations:** 1 Institut Català d’Oncologia (ICO), Cancer Epidemiology Research Program, Barcelona, Spain; 2 Pharmacoeconomics and Outcomes Research Iberia (PORIB), Madrid, Spain; 3 Pathology Department. Hospital, Universitario de Getafe, Madrid, Spain; 4 Gynecology and Obstetrics. Hospital Universitario Basurto, Bilbao, Spain; 5 Pathology Department, Hospital, Universitario Puerta del Mar, Cádiz, Spain

**Keywords:** Cervical cancer screening, Human papilloma virus, Aptima HPV assay, Cost analysis

## Abstract

**Background:**

HPV cervical cancer screening (CCS) must use validated HPV tests based on the molecular detection of either viral mRNA (Aptima HPV Assay—AHPV) or DNA. AHPV has demonstrated the same cross-sectional and longitudinal sensitivity for the detection of HSIL/CIN2+ lesions but with greater specificity than HPV-DNA tests. The study aimed to estimate the total costs of a CCS with a primary HPV test based on the detection of mRNA compared to DNA in women aged 35–65 years for the National Health System.

**Methods:**

A decision-tree-based model to estimate the cost of the CCS until the first colposcopy was designed based on Spanish CCS guidelines. The total cost (€, 2019) for CCS with AHPV or DNA tests (HC2 and Cobas) was calculated, including HPV test, liquid-based cytology (LBC) and colposcopy, for a population of 7,263,529 women aged 35–65 years (assuming 70% coverage). Clinical inputs derived from a literature review were validated by a multidisciplinary expert panel. Data from head-to-head studies between different HPV tests were selected.

**Results:**

The use of AHPV showed reduction of 290,541 (− 35%) and 355,913 (− 40%) LBC compared to HC2 or Cobas, respectively. Furthermore, AHPV avoided 151,699 (− 47%) colposcopies versus HC2 and 151,165 (− 47%) versus Cobas. The total cost of CCS was € 282,747,877 with AHPV, € 322,587,588 with HC2 and € 324,614,490 with Cobas. Therefore, AHPV savings € − 39,839,711 versus HC2 and € − 41,866,613 versus Cobas.

**Conclusions:**

Assuming that 70% of women from 35 to 65 years attend the CCS programme, the cost of screening up to the first colposcopy using AHPV would provide cost savings of up to € 41.9 million versus DNA tests in Spain.

**Supplementary Information:**

The online version contains supplementary material available at 10.1186/s12905-021-01310-8.

## Background

Cervical cancer, with at least 500,000 new cases diagnosed every year and a world standardized incidence of 13.1 per 10^5^ women is the fourth most frequent malignant tumour among women, and the fourth leading cause of cancer death worldwide.

According Globocan estimation in 2018, the standardized mortality is 6.9 per 10^5^ women over the world, meaning that there are approximately 311,000 deaths annually occurred due to cervical cancer. Approximately 85% of these deaths occur in developing countries [1].

In Spain, one of the countries with the lowest incidence of this cancer in the world, the incidence of cervical cancer is around 5.2 × 10^5^ women, and the cervical cancer related mortality is 1.7 cases per 100,000 women per year [1]. In absolute numbers, these figures represent 1900 cervical cancer diagnoses and 825 deaths per year [1].

The aetiological cause of cervical cancer is the infection with oncogenic types of human papillomavirus (HPV) [2, 3]. HPV infection is a sexually transmitted disease, which affects the anogenital and oral areas. It is very common among the sexually active population, reaching the highest prevalence at the beginning of sexual intercourse, with a marked decrease after 30 years of age [4, 5].

Fortunately, the development of vaccines against HPV and the updating of screening guidelines which recommend the replacement of cytology for the HPV test as a primary screening, have made cervical cancer a preventable disease [6]. In fact, the World Health Organization defined the cervical cancer as a worldwide public health concern, 2018 as the beginning year of its elimination, establishing the objective of reducing its annual incidence below 4 cases per 10^5^ women [7].

Traditionally, cervical cancer screening (CCS) has been based on the analysis of conventional or liquid-based cytology (LBC) with the aim of detecting high grade cervical intraepithelial neoplasia or worse (HSIL/CIN2+) lesions to treat them and prevent their progression to cancerous lesions [8]. However, there are several scientific publications which support the superiority of HPV tests as a primary screening in comparison to cytology [6, 9]. HPV tests are associated with an increase in sensitivity of 30–40% for the detection of HSIL/CIN2+ and a loss of 3–5% of specificity [9]. Clinical trials conducted in European countries, for which there are follow-up data of at least two rounds of screening, have shown that primary screening with HPV tests, starting at age 30, could improve 60–70% of increased protection against cervical cancer as compared to cytology-based screening programme [10]. These studies also showed that screening with an HPV test every 5 years offers better protection than screening with cytology every 3 years [10].

Given the extensive accumulated evidence, the main scientific societies involved in the prevention of this cancer have released updated guidelines [6], recommending the implementation of a population-based screening programme and the implementation of HPV screening with a preference over a cytology screening strategy in women above 30 years. On the other hand, it has also been internationally agreed that for its use in screening, only clinically validated HPV detection techniques can be used [11].

The National Health Ministry in Spain recommends that CCS be based on European and National practice guidelines, using two primary tests for the detection of cervical cancer: 1) LBC every 3 years for women aged 25–34 years with an HPV test as triage for women with atypical squamous cells of unknown significance (ASC-US) and colposcopy for the remaining abnormal cytological results, and 2) HPV primary testing every 5 years in women between 35 to 65 years of age with cytology triage in case of HPV-positive result [6, 12–14].

There are several validated HPV tests, available for primary screening, based on the molecular detection of either HPV mRNA or DNA. According to clinical evidence, the Aptima HPV Assay (AHPV), has shown to have the same cross-sectional and longitudinal sensitivity but higher specificity than DNA HPV tests for detecting HSIL/CIN2+ [15–18], when used as a primary screening test. Due to their higher specificity, this test can reduce false-positive results, avoiding unneeded anxiety for women, overdiagnosis and overtreatment and therefore leading to saving costs for health systems [19, 20].

The estimation of the economic benefit derived from the increased test specificity applied to a CCS programme has not yet been assessed and reported. The objective of the present study was to estimate the total costs from the outset up to the first colposcopy of a population-based CCS with a primary HPV test based on the molecular detection of mRNA versus DNA in Spain.

## Methods

### Model structure

A cost-analysis *de novo* model was developed using Microsoft Excel^®^. The design and structure of the model as well as the parameters required for the development of the analysis were defined by a multidisciplinary expert panel composed of 2 gynaecologists, 3 pathologists, 1 epidemiologist and 3 health economics specialists. A structured questionnaire that included the values identified in the scientific published literature was developed and filled by the expert panel. Subsequently, an expert meeting was carried out to validate and agree upon all the values used in the analysis.

### Modelling

Two scenarios were considered to estimate the costs associated with the CCS for all Spanish populations covered by the screening programme, including the first colposcopy. The initial analysis defined as the base case, was performed in women aged 35–65 years, where the primary screening was performed with HPV testing. An alternative analysis (alternative case) considered the subgroup of women 25–34, where the primary screening was carried out with LBC.

**Fig. 1:**
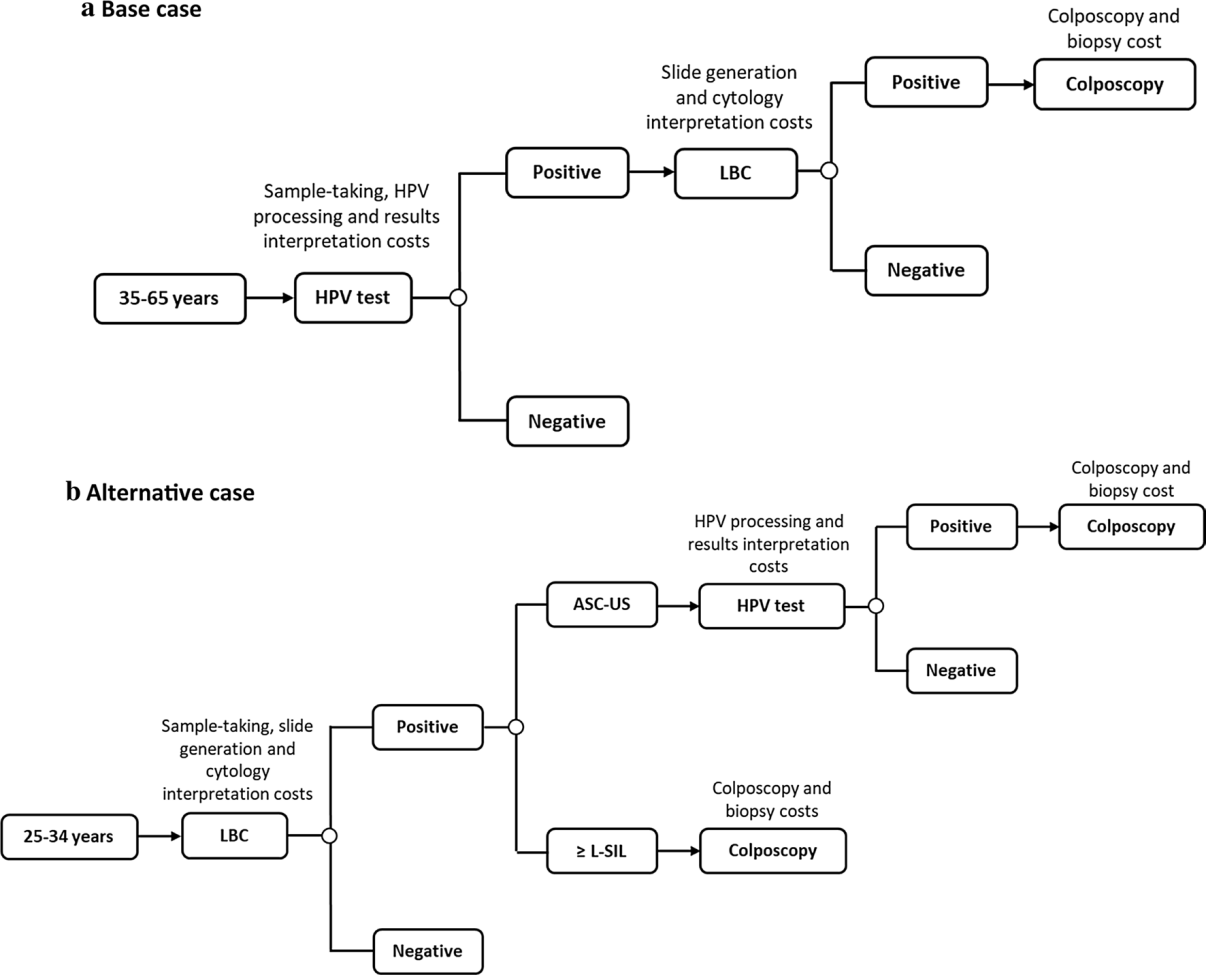
Decision tree model and procedures included. ASC-US: atypical squamous cells of undetermined significance; HPV, human papilloma virus; LBC, liquid-base cytology; L-SIL, low-grade squamous intraepithelial lesions. In both scenarios, LBC was considered in the analysis for sample collection, allowing the reflex test to be performed

According to the recommendations of the current screening guidelines in Spain [13], and due to the clinical feature of the disease, it was decided to represent the evolution of patients by the CCS through a decision tree model, which was designed following the ISPOR recommendations for modelling a good research practices [21], for the two scenarios (Fig. 1).

The analysis started with the cohorts accessing the CCS. Along the time horizon of the simulation, women were transitioning between the different nodes according to the probability of event occurrence. For this economic analysis, the model was stops after performance of the colposcopy in those women requiring it. The decision tree nodes represented events derived from the findings of their HPV, LBC, colposcopy, and biopsy results.

Furthermore, LBC was considered in the analysis for sample collection, allowing the reflex test to be performed in both scenarios, thus avoiding the collection of a new sample.

The probabilities needed for model feeding were obtained from the available scientific epidemiological publications and clinical trials of HPV testing.

### Study population

Following the recommendations of the Spanish guidelines for CCS [13, 22], the primary HPV CCS included the Spanish population of women aged 35–65 years (base case), and the cytological screening included women aged 25–34 (alternative case).

Figures of the different populations were obtained from the National Institute of Statistics for 2018 [23]. It was assumed that all women in the target population would be invited to participate in the CCS. In accordance with the AFRODITA study [24], it was considered that 70% of invited women would attend their screening appointment. Therefore, the final population assessed in the model comprised 7,263,529 Spanish women aged 35–65 years and 1,947,925 women aged 25–34 years, assuming that all of them were asymptomatic.

### HPV testing

Based on the expert panel recommendations, three molecular detection HPV tests were assessed in the present analysis: one HPV mRNA test [Aptima human papillomavirus assay (Hologic, Inc., San Diego, USA)] and two HPV DNA tests [Hybrid Capture 2 high-risk HPV DNA test (Qiagen, Gaithersburg, MD, USA) and Cobas 4800 HPV test (Roche Molecular Diagnostics, Pleasanton, CA, USA)].

The Hybrid Capture 2 (HC2) high-risk HPV DNA test is considered the gold standard of HPV assays, as its performance was validated in many randomized controlled trials. HC2 collectively detects 13 high-risk (hr) HPV genotypes (16, 18, 31, 33, 35, 39, 45, 51, 52, 56, 58, 59 and 68). Aptima human papillomavirus (AHPV) is an in vitro nucleic acid amplification test for the qualitative detection of E6/E7 viral transcript mRNA from 14 h HPV types in cervical smear samples. The hr HPV types detected by the assay are 16, 18, 31, 33, 35, 39, 45, 51, 52, 56, 58, 59, 66, and 68. Likewise, the Cobas 4800 test detects the same 14 h HPV genotypes as the AHPV assay. However, while the Roche assay detects hr HPV DNA, the Aptima assay detects hr HPV oncogenic mRNA expression and is designed to be more specific in identifying clinically significant hr HPV infections that are likely to lead to high-grade squamous dysplasia and neoplasia.

In the model, two different scenarios were compared: scenario 1, AHPV versus HC2; and scenario 2, AHPV versus Cobas 4800. Although some of these HPV tests give, in addition to the overall result for all hr HPV types including (positive/negative) provide the partial result for HPV 16 and HPV 18, this partial result was not considered in this analysis.

### Clinical data

A literature review was conducted in Medline through PubMed to identify scientific publications regarding the clinical evidence available in this field and to extract the probabilities required for the model. Details of the search strategy applied for the literature review is shown in Additional file 1: Appendices 1 and 2.

An initial selection of 1408 localized references was performed by reading the title and abstract. Subsequently, 80 studies were considered relevant for this analysis and were selected and reviewed in full text. The probabilities of the different clinical data included in the analysis were obtained from those clinical studies considered the most relevants. Among all publications, the studies providing data from direct comparisons between the selected HPV tests were preferred and prioritized as sources for inputs.

In this sense, the prevalence of HPV for base case was obtained from a transversal head-to-head study about HPV tests in a screening population [25]. This study showed rates of 7.5%, 11.50% and 12.40% for AHPV, HC2 and Cobas 4800, respectively (Table 1).

**Table 1:** Model inputs

	AHPV versus HC2		AHPV versus Cobas 4800	
	AHPV	HC2	AHPV	Cobas
*Transition probabilities (%)*				
Women 35–65 years				
HPV positive	7.5% [20]	11.5% [20]	7.5% [20]	12.4% [20]
Abnormal LBC after HPV positive	31.1% [21]	38.5% [23]	31.1% [21]	35.6% [22]
Women 25–34 years				
Abnormal LBC	6.5% [21]	6.5% [21]	6.5% [21]	6.5% [21]
ASC-US in patients with abnormal LBC	46.0% [21]	46.0% [21]	46.0% [21]	46.0% [21]
HPV test positive in patients with ASC-US	42.0% [14]	53.0% [14]	53.0% [39]	58.2% [39]
L-SIL + in patients with abnormal LBC	74.1% [40]	74.1% [40]	74.1% [40]	74.1% [40]

	Unit cost (± SD)	References
Unit costs (€, 2019)		
HPV primary test	€ 31.81^b^	[17, 25]
HPV secondary test	€ 21.90^c^	[17]
LBC primary	€ 42.55 (± 12.13)^a,d^	[17, 26]
LBC secondary	€ 32.64 (± 12.13)^a,e^	[26]
Colposcopy and biopsy	€ 200.11 (± 2.73)^a,f^	[24, 26]

ASC-US+: any cytology result from atypical squamous cells of undetermined significance to carcinoma; AHPV: Aptima HPV Assay; HC2: Hybrid Capture 2 Assay; HPV: human papilloma virus; LBC: liquid-base cytology; L-SIL+: any cytology result from low-grade squamous intraepithelial lesions to carcinoma; SD: Standard deviation

^a^Average cost obtained from the list of references identified in the Spanish database

^b^Includes sample-taking costs (human resources, disposables and vial), costs of molecular detection of HPV processing and interpretation of results

^c^Includes costs of molecular detection of HPV processing and interpretation of results; 3. Includes sample-taking costs (human resources, disposables and vial), costs of cytology processing and interpretation

^d^Includes costs of cytology processing and interpretation

^e^Includes the colposcopy procedure with processing and interpretation of one biopsy

Regarding the proportion of women with abnormal LBC after a positive HPV test result, several studies were identified [26–28]. These transition probabilities are shown in Table 1.

For the alternative case, the prevalence of women with abnormal LBC was 6.5% [26]. Other transition probabilities for the different nodes considered in the decision tree model for this subgroup of women aged 25–34 years are shown in Table 1.

### Costs

The analysis was carried out from the perspective of the Spanish National Health System (NHS); therefore, only direct health care costs were included, comprising HPV and diagnostic test costs (LBC, colposcopy, and biopsy costs). Figure 1 describes when and which of each of the health care resources were considered.

Unitary costs were obtained from the available scientific literature or regional public information [22, 29, 30] and from a national database of health care costs [31]. All costs included in the model were expressed in euros for 2019, updating the costs obtained from the literature based on the Spanish general consumer price index, if needed [32].

Table 1 shows the unitary costs of the direct health care resources included in the model.

### Sensitivity analysis

Several sensitivity analyses (SA) were performed to incorporate the uncertainty into the analysis and to observe the effect of these modifications on the results. The following parameters were varied individually: 1) One-way SA was carried out considering a possible reduction in the women from 35 to 65 years of age who would attend their screening appointment, assuming that 36.3% of these women attended the CCS in private practice [24] and that 44.6% of first screening attendees used the public sector. In all cases, an organized CCS with an individual invitation to the target women was considered. 2) To represent a range for the most plausible results of the clinical data regarding women from 35 to 65 years with a positive HPV result, a univariate SA was performed with the studies showing the largest [16, 33] and smallest [34, 35] differences between the test considered in the analysis. 3) Finally, primary LBC, colposcopy and biopsy unitary costs were modified considering the alternative values identified in the literature [22, 36]. An additional scenario was performed with the upper and lower values of these health resources, obtained by applying the standard deviation (SD) to the mean value (Table 1).

## Results

Assuming that 70% of women aged 35–65 years would attend the CCS, when AHPV was used instead of HC2 or Cobas 4800 as the primary test, there was a reduction of 290,541 (35% decrease) and 355,913 LBC samples (40% decrease), respectively. Furthermore, the use of AHPV avoided 151,699 (47% reduction) colposcopies compared to HC2 and 151,165 (47% reduction) compared to Cobas 4800 (Table 2).

**Table 2:** Base-case and sensitivity analysis results

Base-case results (women aged 35–65 years N = 7,263,529 women)					
	AHPV	HC2	Cobas 4800	Difference AHPV versus HC2	Difference AHPV versus Cobas 4800
*Number of procedures performed according to HPV detection technology and assuming cervical cancer screening coverage of 70%*					
HPV	7,263,529	7,263,529	7,263,529	–	–
LBC	544,765	835,306	900,678	− 290,541 (− 35%)	− 355,913 (− 40%)
Colposcopies	169,476	321,175	320,641	− 151,699 (− 47%)	− 151,165 (− 47%)
*Cost of performing the different procedures*					
HPV test cost	€ 231,052,857	€ 231,052,857	€ 231,052,857	–	–
LBC cost	€ 17,781,199	€ 27,264,382	€ 29,398,177	€ − 9,483,263	€ − 11,616,998
Colposcopy and biopsy cost	€ 33,931,900	€ 64,270,348	€ 64,163,515	€ − 30,356,448	€ − 30,249,615
Total cost	€ 282,747,877	€ 322,587,588	€ 324,614,490	€ − 39,839,711	€ − 41,866,613

*Sensitivity analysis results*							
	Base-case value	SA value	Cost form entry in screening until first colposcopy was performed				
			AHPV	HC2	Cobas 4800	Difference AHPV versus HC2	Difference AHPV versus Cobas 4800
Target population (36.3% coverage in private service)	7,263,529 women (Assuming a cervical cancer screening coverage of 70%)	4,626,868 women (Assuming a cervical cancer screening coverage of 44.6%)	€ 180,110,398	€ 205,488,294	€ 206,779,430	€ − 25,377,896	€ − 26,669,032
*Transition probabilities (HPV positive)*							
AHPV versus HC2	AHPV: 7.5%	AHPV: 10.3% [13]	€ 302,047,351	€ 356,017,663	–	€ − 53,970,312	–
		HC2: 15.7% [13]					
	HC2: 11.5%	AHPV: 7.2% [29]	€ 280,680,076	€ 297,913,008	–	€ − 17,232,932	–
		HC2: 8.4% [29]					
AHPV versus Cobas 4800	AHPV: 7.5%	AHPV: 15.3% [12]	€ 336,510,697	–	€ 415,158,005	–	€ − 78,647,307
		Cobas: 24.4% [12]					
	Cobas: 12.4%	AHPV: 12.8% [30]	€ 319,279,024	–	€ 341,214,134	–	€ − 21,935,110
		Cobas 14.6% [30]					
LBC, colposcopy and biopsy costs	Primary LBC: € 42.55	LBC: € 50.56 [31]	€ 295,564,919	€ 345,298,601	€ 347,822,501	€ − 49,733,682	€ − 52,257,582
	Colposcopy + biopsy: € 200.11	Colposcopy + biopsy: € 249.99 [31]					
		LBC: € 18.31 [17]	€ 258,447,168	€ 281,312,441	€ 281,789,684	€ − 22,865,273	€ − 23,342,515
		Colposcopy + biopsy: € 134.64 [17]					

AHPV, Aptima HPV Assay; HC2, Hybrid Capture 2 Assay; HPV, human papilloma virus; LBC, liquid-base cytology

The total cost of CCS, including the performance of the first colposcopy, in women aged 35–65 years resulted € 282,747,877 with AHPV, € 322,587,588 with HC2 and € 324,614,490 with Cobas 4800. Therefore, the savings derived from using AHPV versus a DNA HPV test range between 39.8 and 41.9 million euros (Table 2).

**Fig. 2:**
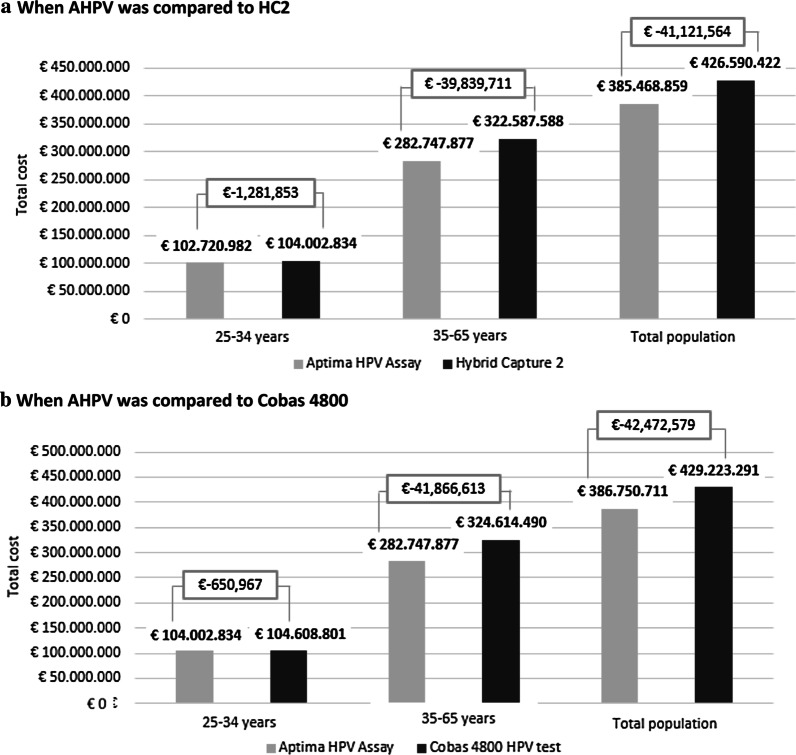
Total cost of cervical cancer screening programme from inclusion to the first colposcopy by age groups. The base case result was described in the columns “35–65 years”, which considers the costs until the first completed colposcopy for women who require it. The other 2 columns describe the results of (1) the alternative case [women aged 25–34 years (columns on the left)] with costs including the first colposcopy and (2) the total cost for the Spanish population of a screening program aged 25–65 years (the last columns on the right, “total population”). In all cases, a 70% population coverage is assumed

Including the activity of CCS in women aged 25–34 years and considering the costs up to the first colposcopy with a 70% coverage, the use of AHPV provided a total reduction of 158,105 colposcopies and 290,541 LBC samples compared to HC2 and 154,193 colposcopies and 355,913 LBC samples versus Cobas 4800. Therefore, the total cost up to, and including the first colposcopy after the CCS programme implementation with AHPV saves up to € − 41,121,564 when compared to HC2 and € − 42,472,579 versus Cobas 4800 (Fig. 2).

### Sensitivity analysis

The results of the different univariate SA are shown in Table 2. AHPV resulted the least costly option and reduced the number of tests performed in all the scenarios, confirming the base case results.

The one-way SA that had the highest influence on the results was considering alternative published evidence of the proportion of women with a positive HPV test result [34, 35], followed by the variation in cost based on literature evidence [22].

When the studies with the greatest differences in HPV positivity were used, AHPV saves up to € − 53,970,312 compared to HC2 and € − 78,647,307 compared to Cobas 4800. On the other hand, if we calculated cost savings based on the studies with the smallest differences in positive HPV results, AHPV showed savings of up to € − 17,232,932 and € − 21,935,110 when compared to HC2 and to Cobas 4800, respectively.

Regarding the reduction in women from 35 to 65 years of age who would attend their screening appointment in the public sector (n = 4,626,868 women), the use of AHPV provided a cost-saving of € − 2,537,896 versus HC2 and € − 26,669,032 versus Cobas 4800.

Based on the decrease in primary LBC, colposcopy and biopsy costs, when the upper value of the cost reported in the literature was applied, using AHPV would save up to € − 49,733,682 compared to HC2 and € − 52,257,582 compared to Cobas 4800 (Table 2). The savings are € − 22,865,273 versus HC2 and € − 23,342,515 versus Cobas 4800 when using the lower value of the reported cost (Table 2).

Regarding the SA calculated with the upper and the lower values obtained by applying the SD to the mean value of primary LBC, colposcopy and biopsy unitary costs, the savings ranged between € − 35,899,792 and € − 43,779,630 for AHPV versus HC2 and between € − 37,135,197 and € − 46,598,028 for AHPV versus Cobas 4800.

## Discussion

Several economic analyses have assessed the cost of a publicly funded CCS in Spain [22, 36–38], all oriented to evaluate the efficiency to implement a HPV test-based primary screening programme in Spain. However, none of these studies evaluated the economic impact of the use of mRNA or DNA tests in the CCS programme. The present study is the first cost-analysis assessing the impact of different HPV tests on overall CCS programme cost by comparing an mRNA test (AHPV) with an HPV DNA test (HC2 or Cobas 4800) in Spain.

The introduction of HPV as primary tests in CCS has the advantage of being more sensitive for the detection of HSIL/CIN2+ but they are substantially less specific than traditional cytology [9]. HPV positive results require to be triaged to differentiate those women with increased risk of having or developing HSIL/CIN2+ lesions from those at lower risk affected with potentially temporal HPV infections. Clinical sensitivity for the detection of HSIL/CIN2+ of the different HPV tests have shown to be similar, with varying overall positivity and clinical specificity. Several studies have demonstrated that AHPV is suitable as a primary screening test for CCS [39, 40], having a similar longitudinal sensitivity and negative predictive value as HPV-DNA-based assays for the detection of HSIL/CIN2+ but with higher specificity [19, 34, 40–42]. The reason is that AHPV detects the expression of HPV E6/7 mRNA, reducing the detection of transient infections that are less likely to progress to more severe lesions as HSIL/CIN2+.

The results of this study supported that the use of AHPV could be associated with a reduction in additional LBC-based triage tests and follow-up procedures, measured by number of colposcopies, with a consequent reduction in the total cost of CCS programmes in 35–65-year-old Spanish women as observed in the alternative scenario. These cost savings are a direct result of the increased specificity reducing the number of women referred for further management.

Similar economic studies have been performed in other countries. In 2012, Sauter JL, et al. (2014) reported a 21% reduction in colposcopy referrals in the 12 months following the change from HC2 to AHPV in women with an ASC-US diagnosis [17]. In our analysis, this reduction is even larger with up to 47% fewer colposcopies, due to the use of HPV as the primary screening test. Two additional US studies have evaluated the economic impacts of AHPV use compared to DNA tests, either with LBC co-testing or as primary HPV testing [43, 44]. These studies similarly report that mRNA assays provide cost savings versus DNA testing. Finally, a recent analysis conducted in England suggested that the use of AHPV over DNA based testing could result in savings of up to £11.3 million (€ 13.8 million) for the screening system [45, 46].

The present model has some limitations. First, the parameters used in the analysis have been extracted from different sources. However, all parameters are based on official sources or on publications with a high level of clinical evidence, and values were validated by a multidisciplinary expert panel. The potential uncertainty associated with some of the parameters was tested in a univariate SAs. Second, the influence of the additional procedure costs performed in women with a positive HPV test result (LCB, colposcopy and biopsy) was also tested in a SA. As observed, none of these two SAs changed the conclusions of the base case results.

Another possible limitation could be related to the use of clinical data extracted from studies conducted in other countries, as no robust head-to-head comparative studies have been conducted in Spain. The literature review provided a wide variety of reports; however, a limited number of studies with direct comparisons between mRNA and DNA tests were found. Among all available studies, two of them [17, 25] evaluated the tests included in this analysis. For our study, these reports were selected to avoid the potential bias associated with differences in populations, methodologies and/or local patient management between different locations. The SA performed using the most plausible values for HPV-positive women (minimum and maximum differences between tests) also maintained the conclusions of the base case.

This study used an analytic model to assess the health cost after a CCS programme implementation (from the beginning of the implementation to the end of the first colposcopy) with a HPV mRNA test (AHPV) compared to an HPV DNA test (HC2 or Cobas 4800) in women aged 35–65 years in Spain. Initially, the decision tree model was developed and designed considering the entire time horizon for a complete CCS programme based on current recommendations from national and European guidelines [6, 12] and was validated and agreed upon by a multidisciplinary expert panel. Due to the lack of reliable clinical data to feed all the probabilities of the model and in order to simplify the analysis, it was decided to shorten the time horizon until the completion of the first colposcopy. The scarce available evidence suggests the need for future development of epidemiological studies that could provide more detailed data to replicate the present analysis with a longer time horizon or follow up after an abnormal test result.

Despite the limitations described above, the results of the SA confirmed that the uncertainty associated with the parameters used in this analysis did not represent a significant deviation from the results obtained in the base case, showing that AHPV is the least costly option, reducing testing in all the scenarios evaluated.

## Conclusion

In conclusion, assuming that 70% of women from 35 to 65 years attend the population-based CCS programme in Spain, the cost of screening up to completion of the first colposcopy using AHPV could generate health cost savings up to 39.8–41.9 million euros when compared to DNA testing.

## Supplementary Information

**Additional file 1. Appendix 1**: Search strategy in PubMed database for LBC. This appendix shows the search strategy carried out in the PubMed database to obtain LBC-related transition probability data to feed the model. **Appendix 2**: Search strategy in PubMed database for HPV test. This appendix shows the search strategy carried out in the PubMed database to obtain VPH test-related transition probability data to feed the model.

## Data Availability

The datasets used and/or analysed during the current study are available from the corresponding author upon reasonable request and with permission of Hologic Spain.

## Abbreviations

AHPV: Aptima HPV Assay
ASC-US: Atypical squamous cells of unknown significance
CCS: Cervical cancer screening
HC2: Hybrid Capture 2 HC2
HSIL/CIN2+: High grade cervical intraepithelial neoplasia or worse
HPV: Human papillomavirus
LBC: Liquid-base cytology
NHS: National Health System
SA: Sensitivity analyses
SD: Standard deviation
