# Supplementary material for: Comparative cost analysis of cervical cancer screening programme based on molecular detection of HPV in Spain
Source: BMC Womens Health. 2021 Apr 26;21:178. doi: 10.1186/s12905-021-01310-8 (PMC8074415; doi:10.1186/s12905-021-01310-8)
Supplement: Supplementary file 1 — Additional file 1. Appendix 1: Search strategy in PubMed database for LBC. This appendix shows the search strategy carried out in the PubMed database to obtain LBC-related transition probability data to feed the model. Appendix 2: Search strategy in PubMed database for HPV test. This appendix shows the search strategy carried out in the PubMed database to obtain VPH test-related transition probability data to feed the model. [file 12905_2021_1310_MOESM1_ESM.docx]

**Appendix**

**Appendix 1. Search strategy in PubMed database for LBC**

|  | Terminology | Searching strategy | No. of references |
| --- | --- | --- | --- |
| 1 | Cervica cytology | cervical cytology[title] | 1.331 |
| 2 | Gynecological cytology | gynecology cytology[title] | 958 |
| 3 |  | gynaecological cytology[title] | 20 |
| #1 OR #2 OR #3 | | | **2.021** |
| 4 | L-SIL | ("squamous intraepithelial lesions of the cervix"[MeSH Terms] OR ("squamous"[All Fields] AND "intraepithelial"[All Fields] AND "lesions"[All Fields] AND "cervix"[All Fields]) OR "squamous intraepithelial lesions of the cervix"[All Fields] OR ("low"[All Fields] AND "grade"[All Fields] AND "squamous"[All Fields] AND "intraepithelial"[All Fields] AND "lesion"[All Fields]) OR "low grade squamous intraepithelial lesion"[All Fields]) | 3.259 |
| 5 | H-SIL | "squamous intraepithelial lesions of the cervix"[MeSH Terms] OR ("squamous"[All Fields] AND "intraepithelial"[All Fields] AND "lesions"[All Fields] AND "cervix"[All Fields]) OR "squamous intraepithelial lesions of the cervix"[All Fields] OR ("high"[All Fields] AND "grade"[All Fields] AND "squamous"[All Fields] AND "intraepithelial"[All Fields] AND "lesion"[All Fields]) OR "high grade squamous intraepithelial lesion"[All Fields] | 3.529 |
| 6 | Carcinoma | cell carcinoma[title/abstract] OR squamous cell carcinoma[title/abstract] | 139.610 |
| 7 | Colposcopy | "colposcopy"[MeSH Terms] | 6.066 |
| 8 | CIN1 | (("cervical intraepithelial neoplasia"[MeSH Terms] OR ("cervical"[All Fields] AND "intraepithelial"[All Fields] AND "neoplasia"[All Fields]) OR "cervical intraepithelial neoplasia"[All Fields]) AND grade[All Fields] AND 1[All Fields]) | 3.503 |
| 9 | CIN2 | (("cervical intraepithelial neoplasia"[MeSH Terms] OR ("cervical"[All Fields] AND "intraepithelial"[All Fields] AND "neoplasia"[All Fields]) OR "cervical intraepithelial neoplasia"[All Fields]) AND grade[All Fields] AND 2[All Fields]) | 3.787 |
| 10 | CIN3 | (("cervical intraepithelial neoplasia"[MeSH Terms] OR ("cervical"[All Fields] AND "intraepithelial"[All Fields] AND "neoplasia"[All Fields]) OR "cervical intraepithelial neoplasia"[All Fields]) AND grade[All Fields] AND 3[All Fields]) | 3.587 |
| 11 | CIN1 OR CIN2 OR CIN3 | CIN1[All Fields] OR CIN2[All Fields] OR CIN3[All Fields] | 2.376 |
| (#1 OR #2 OR #3) AND (#4 OR #5 OR #6 OR #7 OR #8 OR #9 OR #10 OR #11) | | | **669** |

**Appendix 2. Search strategy in PubMed database for HPV test**

|  | | Terminology | Searching strategy | No. of references |
| --- | --- | --- | --- | --- |
| A | | Hybrid capture 2 | ("chimera"[MeSH Terms] OR "chimera"[All Fields] OR "hybrid"[All Fields]) AND capture[All Fields] AND 2[All Fields] | 1.675 |
| B | | Realtime high-risk | realtime[All Fields] AND high-risk[All Fields] | 71 |
| C | | Cobas 4800 | cobas[All Fields] AND 4800[All Fields] | 174 |
| D | | Onclarity | onclarity[All Fields] | 18 |
| E | | Aptima HPV | aptima[All Fields] AND hpv[All Fields] | 83 |
| #A OR #B OR #C OR #D OR #E | | | | **1.916** |
| F | | L-SIL | ("squamous intraepithelial lesions of the cervix"[MeSH Terms] OR ("squamous"[All Fields] AND "intraepithelial"[All Fields] AND "lesions"[All Fields] AND "cervix"[All Fields]) OR "squamous intraepithelial lesions of the cervix"[All Fields] OR ("low"[All Fields] AND "grade"[All Fields] AND "squamous"[All Fields] AND "intraepithelial"[All Fields] AND "lesion"[All Fields]) OR "low grade squamous intraepithelial lesion"[All Fields]) | 3.248 |
| G | | H-SIL | "squamous intraepithelial lesions of the cervix"[MeSH Terms] OR ("squamous"[All Fields] AND "intraepithelial"[All Fields] AND "lesions"[All Fields] AND "cervix"[All Fields]) OR "squamous intraepithelial lesions of the cervix"[All Fields] OR ("high"[All Fields] AND "grade"[All Fields] AND "squamous"[All Fields] AND "intraepithelial"[All Fields] AND "lesion"[All Fields]) OR "high grade squamous intraepithelial lesion"[All Fields] | 3.513 |
| H | | Carcinoma | cell carcinoma[title/abstract] OR squamous cell carcinoma[title/abstract] | 139.067 |
| I | | Colposcopy | "colposcopy"[MeSH Terms] | 6.072 |
| J | | CIN1 | (("cervical intraepithelial neoplasia"[MeSH Terms] OR ("cervical"[All Fields] AND "intraepithelial"[All Fields] AND "neoplasia"[All Fields]) OR "cervical intraepithelial neoplasia"[All Fields]) AND grade[All Fields] AND 1[All Fields]) | 3.488 |
| K | | CIN2 | (("cervical intraepithelial neoplasia"[MeSH Terms] OR ("cervical"[All Fields] AND "intraepithelial"[All Fields] AND "neoplasia"[All Fields]) OR "cervical intraepithelial neoplasia"[All Fields]) AND grade[All Fields] AND 2[All Fields]) | 3.768 |
| L | | CIN3 | (("cervical intraepithelial neoplasia"[MeSH Terms] OR ("cervical"[All Fields] AND "intraepithelial"[All Fields] AND "neoplasia"[All Fields]) OR "cervical intraepithelial neoplasia"[All Fields]) AND grade[All Fields] AND 3[All Fields]) | 3.571 |
| M | | CIN1 OR CIN2 OR CIN3 | CIN1[All Fields] OR CIN2[All Fields] OR CIN3[All Fields] | 2.356 |
| (#A OR #B OR #C OR #D OR #E) AND (#F OR #G OR #H OR #I OR #J OR #K OR #L OR #M) | | | | **743** |
| N | VPH | | "papillomaviridae"[MeSH Terms] OR "papillomaviridae"[All Fields] OR ("human"[All Fields] AND "papilloma"[All Fields] AND "virus"[All Fields]) OR "human papilloma virus"[All Fields] | 33.091 |
| (#A OR #B OR #C OR #D OR #E) AND (#F OR #G OR #H OR #I OR #J OR #K OR #L OR #M OR #N) | | | | **1.136** |
